# Supplementary material for: A comprehensive and quantitative comparison of text-mining in 15 million full-text articles versus their corresponding abstracts
Source: PLoS Comput Biol. 2018 Feb 15;14(2):e1005962. doi: 10.1371/journal.pcbi.1005962 (PMC5831415; doi:10.1371/journal.pcbi.1005962)
Supplement: S1 Table — (DOCX) [file pcbi.1005962.s007.docx]

Table 1: The top 15 journals in the corpora.

| Journal name | Articles published |
| --- | --- |
| *Lancet* | 395,324 |
| *PLoS ONE* | 172,002 |
| *Tetrahedron Letters* | 88,782 |
| *Biochemical and Biophysical Research Communications* | 74,730 |
| *Brain Research* | 60,051 |
| *Chemical Physics Letters* | 57,614 |
| *Journal of Chromatography a* | 54,535 |
| *Physics Letters. B* | 54,255 |
| *Febs Letters* | 53,140 |
| *International Journal of Rock Mechanics and Mining Sciences and Geomechanics Abstracts* | 52,170 |
| *Journal of the Franklin Institute* | 51,926 |
| *Journal of the American College of Cardiology* | 50,992 |
| *Journal of Urology* | 47,370 |
| *Fuel and Energy Abstracts* | 46,055 |
| *American Journal of Cardiology* | 45,073 |
