# Supplementary material for: A comprehensive and quantitative comparison of text-mining in 15 million full-text articles versus their corresponding abstracts
Source: PLoS Comput Biol. 2018 Feb 15;14(2):e1005962. doi: 10.1371/journal.pcbi.1005962 (PMC5831415; doi:10.1371/journal.pcbi.1005962)
Supplement: S2 Table — (DOCX) [file pcbi.1005962.s008.docx]

Table 2: True Positive Rate at 10% False Positive Rate (TPR@10%FPR) for the four different corpora, with and without document weight for scoring co-occurrences.

| Disease-gene associations | \| Source \| TPR@10%FPR,  +document weight \| TPR@10%FPR,  -document weight \| \| --- \| --- \| --- \| \| All Fulltexts \| 0.25 \| 0.21 \| \| Core Abstracts \| 0.16 \| 0.17 \| \| Core Fulltexts \| 0.23 \| 0.20 \| \| MEDLINE \| 0.20 \| 0.20 \| |
| --- | --- | --- | --- | --- | --- | --- | --- | --- | --- | --- | --- | --- | --- | --- | --- | --- |
| Protein-protein associations | \| All Fulltexts \| 0.11 \| 0.09 \| \| --- \| --- \| --- \| \| Core Abstracts \| 0.11 \| 0.10 \| \| Core Fulltexts \| 0.11 \| 0.09 \| \| MEDLINE \| 0.10 \| 0.10 \| |
| Protein-compartment associations | \| All Fulltexts \| 0.04 \| 0.04 \| \| --- \| --- \| --- \| \| Core Abstracts \| 0.04 \| 0.04 \| \| Core Fulltexts \| 0.04 \| 0.04 \| \| MEDLINE \| 0.05 \| 0.05 \| |
